# Pedicle Screw Placement in Adolescent Idiopathic Scoliosis: A Comparison between Robotics Coupled with Navigation versus the Freehand Technique

**DOI:** 10.3390/s22145204

**Published:** 2022-07-12

**Authors:** Gabriel S. Linden, Semhal Ghessese, Danielle Cook, Daniel J. Hedequist

**Affiliations:** 1 Department of Orthopaedic Surgery, Boston Children’s Hospital, Boston, MA 02115, USA; gabriel.linden@childrens.harvard.edu (G.S.L.); semhal.ghessese@childrens.harvard.edu (S.G.); danielle.cook@childrens.harvard.edu (D.C.); 2 Harvard Medical School, Boston, MA 02115, USA

**Keywords:** robotics, AIS, navigation, scoliosis, pediatrics

## Abstract

(1) Background: Robotics coupled with navigation (RAN) is a modern surgical platform shown to increase screw placement accuracy during pediatric scoliosis surgery. Our institution uses a technique which combines the RAN platform for apical pedicle screw placement and the freehand (FH) technique for terminal pedicle screw placement during scoliosis surgery (termed hybrid technique). We question if the complementary use of the RAN technology affects intraoperative outcomes, relative to the FH-only approach. (2) Methods: 60 adolescent idiopathic scoliosis (AIS) patients, ages 11–19 at surgery, who were operated on from 2019 through 2020 by a single surgeon, were retrospectively reviewed. Patients were separated by surgery type (hybrid RAN or FH), matched on demographic and surgical factors, and their intraoperative outcomes were compared statistically. (3) Results: Hybrid RAN patients had more screws placed (*p* = 0.01) and were of a higher BMI percentile (*p* = 0.005). Controlling for the number of screws placed, BMI%, and initial curve magnitude, there were no statistical differences in estimated blood loss per screw (*p* = 0.51), curve correction (*p* = 0.69), complications (*p* = 0.52), or fluoroscopy time (*p* = 0.88), between groups. However, operative time was two minutes longer per screw for hybrid RAN patients (*p* < 0.001). (4) Conclusions: Hybrid RAN surgeries took longer than FH, but yielded comparable effectiveness and safety as the FH technique during the initial RAN adoption phase.

## 1. Introduction

The surgical treatment of adolescent idiopathic scoliosis (AIS) has evolved over the last twenty years to include the use of segmental pedicle screw fixation to enhance curve correction and improve quality of life scores [1]. However, the effectiveness of this technique depends on accurate—and safe—pedicle screw placement. When screw placement goes awry, the resulting complications can be serious. The risks of pedicle screw malposition include neurologic injury, visceral injury, and the potential need for revision surgery [2,3,4]. Original descriptions of freehand (FH) placement of pedicle screws for AIS revealed breaches in the pedicle anatomy in up to 10–15 percent of pedicles [1]. Malpositioned implant placement remains the most common type of instrument-related complication for scoliosis surgery, and thus is a major cause of concern for patients and spinal deformity surgeons [5].

To prevent aberrant pedicle screw placement, technologies such as computer-assisted navigation (CAN) and robotics emerged. CAN has been shown to improve screw placement accuracy, while reducing re-operation incidence in multiple studies [6,7]. CAN allows the surgeon to visualize the patient’s bone anatomy during anatomical registration and uses instruments to create a path for pedicle screws, but is still subject to human error and surgeon tremor. Robotic surgical technology evolved to address problems in CAN and FH related to surgeon manual error. The original robotic system—Mazor (Spine-Assist)—demonstrated that robotically placed pedicle screws were clinically acceptable in 98% of 840 cases [8]. Further advancement of technology allowed for preoperative planning programs based on anatomical registration coupled with a robotic arm to guide the surgical trajectory of instruments. One inherent difficulty with this robotic technology, however, was a lack of tactile feedback during implant placement, as well as a lack of visual confirmation of the anatomy, such as with CAN.

In 2019, the Food and Drug Administration (FDA) cleared the Mazor X Stealth system (Medtronic; Minneapolis, MN, USA) for clinical use in the United States, which couples robotics with CAN (RAN). Myriad research studies demonstrate that RAN pedicle screw placement is highly accurate in adults; however, there is a lack of data reporting on the safety of RAN screw insertion in the pediatric spine population. Gonzalez et al. (2020) reported on the surgical accuracy of RAN screw placement in the pediatric population [9]. This study reinforced previous findings suggesting that RAN accuracy was high—98.7% accurate—even in children. While these results validate RAN as an accurate method for screw insertion in pediatric patients, they do not provide insights on the initial intraoperative outcomes, safety, and efficiency of RAN for surgeons interested in adopting novel robotic techniques into their spine practice. To expand on previous findings about the use of RAN in AIS populations, this series reports on the differences in intraoperative outcomes, safety, and efficiency between two cohorts of AIS patients who either had an FH-only surgery, or a hybrid RAN procedure. This study also reports on the learning curve of RAN, analyzing whether surgical outcomes or efficiency changed over time as the senior author acclimated to RAN.

## 2. Materials and Methods

*Cohort.* Institutional Review Board approval was obtained for this study. AIS patients who received posterior spinal fusion at a single center by the senior author from January 2019 to November 2020 were retrospectively reviewed. Retrospective data were gathered only from patients who had previously provided informed consent into a prospective registry. Patients had pedicle screws placed either by the FH method or by a hybrid RAN technique. The hybrid technique encompasses both RAN and FH, where screws are placed robotically over one registered area encompassing the apical five to seven vertebral levels, and remaining screws outside those segments were placed via the FH technique. Otherwise, the remaining surgery was identical between RAN and FH.

**Figure 1:**
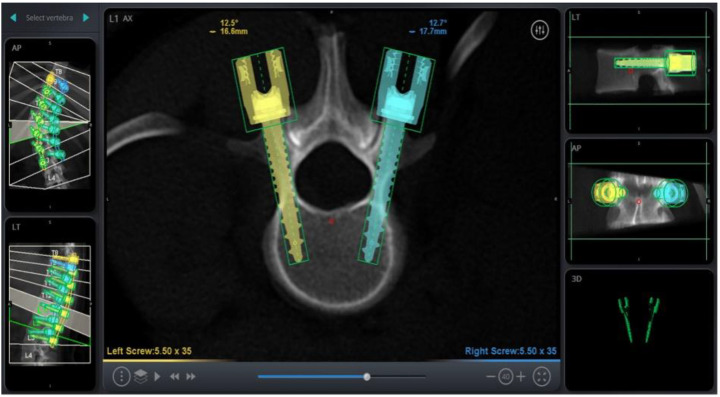
Screw Trajectory Planning on RAN Platform.

**Figure 2:**
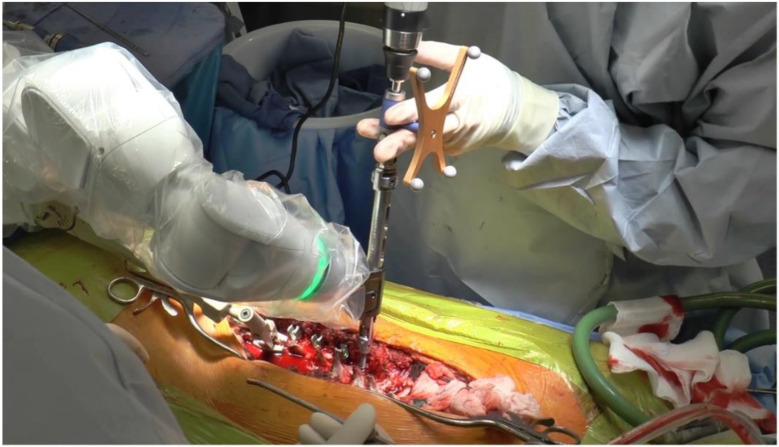
Pedicle Screw Placement Through the Robotic Arm using Navigation.

RAN technology relies on three-dimensional imaging and computer software to plan appropriate pedicle screw placement based on the patient’s anatomy (Figure 1). Once the patient’s anatomy is registered to the system, the robotic arm is sent to each screw placement trajectory location at the selected vertebral levels. Instruments are then sent through the robotic arm to guide placement, with navigation confirming positions in real time (Figure 2).

Intraoperative imaging was obtained to ensure implant placement safety, either using an O-arm, C-arm, or both technologies. Fluoroscopy was not used to guide implant insertion during pedicle screw placement, but rather to ensure safe implant positioning post-placement. Fluoroscopy radiation time was compared between surgical types as the O-arm is not used for FH cases. Pediatric radiation exposure protocols for intraoperative imaging are followed at our institution, which are consistent with published literature [10]. There was no variation between surgical teams for both procedures. Demographic information, intraoperative outcomes, surgical characteristics and immediate postoperative metrics were collected for both surgical groups. Major curves were defined using the Lenke classification system, where curves with the greatest magnitude and structure were considered major [11]. Postoperative variables included complication incidence and type, as well as immediate curve correction.

*Statistical Testing.* An initial power analysis was performed which demonstrated a cohort size of 30 patients per group would be sufficient for the planned data analysis. Patient characteristics and surgery characteristics were summarized and displayed in tabular form. Continuous variables were summarized by mean and standard deviation (SD) or median and interquartile range (IQR), as appropriate, while categorical variables were summarized by frequency and percent. The cohort was separated by surgery type, and comparisons between hybrid RAN and FH pedicle screw placement were conducted using Student’s t-tests, Mann–Whitney U-tests and chi-square tests, where appropriate. Multiple regression analysis was conducted to determine the association between surgery type and outcomes. The number of screws placed, initial curve magnitude, and BMI percentile were controlled for in each multivariable model. Multivariable logistic regression was also utilized to determine if there were any significant associations between surgery type and postoperative complications, controlling for number of screws placed, BMI percentile, fluoroscopy time, and initial curve magnitude. Pearson correlation coefficients were calculated between days from initial robotic surgery and operative time, radiation exposure, and estimated blood loss per level fused. *p*-values < 0.05 were considered significant. Statistical Analysis Software (SAS version 9.4, SAS Institute, Cary, NC, USA) was used.

## 3. Results

Sixty patients were included in the cohort, of which 30 patients had a hybrid RAN surgery (50%) and 30 patients had an FH-only surgery (50%). The mean age at surgery for the cohort was 15.2 years, and 77% were female (Table 1). The thoracic region was the most frequent major curve region (77%), and the mean initial curve magnitude was 63.2 degrees (range, 31.0 to 113.0 degrees). Mean age and initial curve magnitude were comparable for both surgery groups (Table 1), and there were 23 females in both groups (77%; *p* = 1.00). However, the FH group had a significantly higher BMI percentile compared to the hybrid RAN surgery group (80% vs. 49%; *p* = 0.005). Patients who had hybrid RAN surgery had more levels fused on average (10 vs. 7.5, *p* = 0.02). There was a significant difference in the number of screws placed for each surgery type. The hybrid RAN group had a median of 17 screws placed per surgery, and the FH group had a median of 13 screws placed per surgery (*p* = 0.01). However, there was no significant difference in screw density between groups (1.74 screws per level for both groups, *p* = 0.71). The median percentage of screws placed using the RAN system in the hybrid RAN group was 53%.

The median operative time per screw for the hybrid RAN group was 14 min compared to 12 min in the FH surgery group (*p* = 0.001). This value was obtained by dividing the total operative time by the number of screws placed and is not a direct recording of screw placement time. Fluoroscopy time per screw was comparable between the two groups, as both the hybrid RAN group and the FH group were exposed for 0.02 min per screw (Table 2). Additionally, there was no significant difference detected between the two groups for median estimated blood loss per screw (*p* = 0.51).

Patients in the hybrid RAN group had an operative time that was 55 minutes longer compared to the freehand group (β = 54.9; SE = 14.8; *p* < 0.001), controlling for the number of screws placed, BMI percentile, and initial curve magnitude. However, there were no association detected between surgery type and fluoroscopy exposure (*p* = 0.38), EBL (*p* = 0.89), length of stay (*p* = 0.60), curve correction (*p* = 0.69), or postoperative complications (*p* = 0.45). Hybrid RAN surgeries were conducted over an 18-month period for this study. There were no significant correlations found between time from first RAN surgery and operative time (*r* = −0.07, *p* = 0.58), fluoroscopy time (*r* = 0.23, *p* = 0.07) or estimated blood loss per screw (*r* = −0.07, *p* = 0.59).

## 4. Discussion

The treatment of AIS has evolved to include pedicle screw fixation in the thoracic and lumber spine in order to maximize curve correction and patient outcomes. Pedicle screw fixation is a powerful technique; however, it is still fraught with the potential for screw malposition and associated neurologic deficit, visceral injury, and re-operation [3,12]. Technologies such as robotics and computer-assisted navigation (CAN) evolved to increase screw placement accuracy, relative to the traditional FH method, but each system has drawbacks—robotics has a lack of tactile feedback during implant placement, and CAN is dependent on the movements of the surgeon’s hand, which are subject to tremor. Coupling of navigation with robotics (RAN) has been the next step in enabling technologies of screw placement. These platforms allow for preoperative planning, a robotic arm for screw placement, and computer-assisted navigation linked to the robotic arm for real time confirmation of appropriate trajectories [9]. In addition to RAN, virtual augmented reality (AR) is another enabling technology becoming popular in spine surgery [13]. However, this system has not yet been coupled with RAN, making it of less relevance to the technique documented in this study. Nevertheless, AR combined with RAN may be the logical next step as technologies continue to evolve in the pursuit of increased spine surgical safety.

Patients in this series were treated with FH screw placement or a hybrid RAN technique. The goal of this study was to determine what effect the adoption of this technology had on initial intraoperative outcomes, safety, and operative efficiency, relative to traditional methods. The findings in this series suggest that intraoperative outcomes between both groups were comparable, as curve correction, EBL, and length of stay were not statistically different (Table 2). Though curve correction was similar between groups, this measure is not solely dependent on screw placement; there are other maneuvers involved during surgery which also influence curve correction. However, neither technique prohibited this process. Two patients in the FH group experienced a postoperative complication, but this sample was not large enough to detect a statistical difference between surgery types and complication incidence. Of these patients, one was readmitted to the hospital on post-operative day five for postural headaches. The patient had a normal magnetic resonance imaging and computer tomography showed containment of all screws. All symptoms resolved following a non-directed epidural blood patch treatment. The other patient was readmitted for a syncopal episode after surgical discharge. After readmission and evaluation, the syncopal episodes resolved.

Operative time was longer for the hybrid RAN technique than the FH (*p* < 0.001). The increased operative time for RAN is twofold. First, there is an extra step in RAN to register the software to the patient’s anatomy. The use of RAN at our institution requires an O-arm scan (three-dimensional imaging) of the spine segment being instrumented, and then screw placement planning at each level. All of the patients in the RAN group had one O-arm registration. While there is likely intraoperative variability in the amount of time needed to register the patient’s anatomy and plan screw placement, the findings from the senior author’s experience suggest it takes anywhere from 15 to 20 minutes per region. The second factor related to increased time in RAN is preparation of the docking site for the drill guide used for screw placement. “Skiving” of the drill bit has been a noted problem in robotics and is related to abnormal surface topography at the point of drill trajectory. If drill slippage occurs, accurate screw placement may be compromised. To reduce the chance for skiving, preparation of the entry site may be improved by burring away uneven bone surfaces to ensure the drill cannula sits flush against the entry point. This step, while important, needs to be done at every entry point and thus adds time per vertebral segment. The senior author currently has changed this practice with the addition of a high speed navigated drill (Mazor Midas) which runs at approximately 75,000 revolutions per minute to reduce time spent during this surgical step.

This series reports on our initial experience with the RAN platform, but we recognize there may be some limitations to this study. As this study was retrospective, perfect randomization of patients into a hybrid RAN or FH treatment arm before surgery was not possible. The decision to use RAN was multifactorial, and depended largely on the availability of a RAN-trained surgical team and the robotic platform itself over the time period in this study. The robot was not used in the following scenarios: at times where another robotic case was occurring, on Saturdays when the surgical team was unfamiliar with the platform, on days when there were two AIS cases, and during the afternoons. This is an institutional limitation to RAN that surgeons should anticipate prior to adoption of the platform. Likewise, the average BMI of patients in the FH group was higher than that of the RAN group. BMI percentile was controlled for in our multivariable model, ensuring accurate comparison of surgical outcomes between groups. But there was certainly heterogeneity between our groups, as documented, which should be considered when evaluating the surgical differences between cohorts in this study.

For surgeons interested in the adoption of RAN technology, this series suggests that the hybrid RAN technique yields comparable intraoperative effectiveness and safety compared to the gold-standard FH method for operative treatment of AIS. From the first RAN surgery to the last in this report, there were no statistically significant changes in operative time, fluoroscopy exposure, or EBL, suggesting that the initial acclimation to this technology did not sacrifice patient safety. The similarity between outcomes and safety between both methods further supports that an FH-trained surgeon can adopt this hybrid RAN technique and use it effectively. Despite an increase in operative time—which did not affect patient EBL, length-of-stay, complication incidence, or general safety—RAN represents a novel technology that can be used complementary to the FH technique to yield effective curve correction, safety, and enhanced screw placement accuracy for patients with AIS.

## Figures and Tables

**Table 1:** Summary of cohort demographics (n = 60).

	Full Cohort (n = 60)		Hybrid RAN Group (n = 30)		FH Group (n = 30)		
**Characteristic**	**Freq.**	**(%)**	**Freq.**	**(%)**	**Freq.**	**(%)**	***p*** **-Value**
Age at surgery (*years; mean* ± *SD*)	15.2	±1.94	15	±2.01	15.3	±1.9	0.64
Sex (*% female*)	46	(77%)	23	(77%)	23	(77%)	1.00
Race (n = 42) *							
White	36	(60%)	15	(50%)	21	(70%)	
Black or African American	3	(5%)	2	(7%)	1	(3%)	
Other	3	(5%)	2	(7%)	1	(3%)	
Hispanic	6	(10%)	2	(7%)	4	(13%)	0.55
BMI percentile (*median (IQR); n = 59*) *	64	(35–86)	49	(25–70)	80	(49–89)	0.005
Initial major curve magnitude	63.2°	±13.12°	66.4°	±15.29°	60°	±9.77°	0.06
Number of screws	15	(13–19)	17	(14–19)	13	(12–15)	0.01
Major curve region *(apex)*							0.47
Thoracic *(T2-T11/12 Disc)*	46	(77%)	21	(70%)	25	(83%)	
Thoracolumbar *(T12-L1)*	7	(12%)	4	(13%)	3	(10%)	
Lumbar *(L1/2 Disc–L4)*	7	(12%)	5	(17%)	2	(7%)	

SD, standard deviation; IQR, interquartile range. * The number in parentheses represents the number of cases with available data for the given characteristic.

**Table 2:** Summary of cohort outcomes and comparisons between surgery groups (n = 60).

	Full Cohort (N = 60)		Robotic Group (n = 30)		Freehand Group (n = 30)		
**Characteristic**	**Median**	**(IQR)**	**Median**	**(IQR)**	**Median**	**(IQR)**	***p*-Value**
Operative time (min)	202	(161–234)	232	(205–269)	164	(139–193)	<0.001
Operative time per screw (min per screw)	13	(11–15)	14	(13–17)	12	(11–14)	0.001
EBL (cc)	150	(100–300)	200	(106–375)	150	(100–238)	0.21
EBL per screw (cc per level)	12	(8–17)	12	(8–20)	11	(8–15)	0.51
Fluoroscopy time (min)	0.2	(0.2–0.4)	0.3	(0.2–0.4)	0.2	(0.2–0.3)	0.51
Fluoroscopy time per screw (min per screw)	0.02	(0.01–0.03)	0.02	(0.01–0.03)	0.02	(0.01–0.02)	0.88
Length of stay (d)	3	(3–4)	3	(3–4)	3	(3–4)	0.86
Initial curve correction (%)	59	(51–65)	61	(50–65)	58	(51–65)	0.62

IQR, interquartile range; EBL, estimated blood loss.

## Data Availability

Not applicable here.
